# Completing the family of human Eps15 homology domains: Solution structure of the internal Eps15 homology domain of γ‐synergin

**DOI:** 10.1002/pro.4269

**Published:** 2022-01-12

**Authors:** Michael Kovermann, Ulrich Weininger, Christian Löw

**Affiliations:** ^1^ Department of Chemistry University of Konstanz Constance Germany; ^2^ Konstanz Research School Chemical Biology KoRS‐CB University of Konstanz Constance Germany; ^3^ Institute of Physics, Biophysics Martin‐Luther‐University Halle‐Wittenberg Halle (Saale) Germany; ^4^ Centre for Structural Systems Biology (CSSB) Hamburg Germany; ^5^ Molecular Biology Laboratory (EMBL) Hamburg Unit c/o Deutsches Elektronen Synchrotron (DESY) Hamburg Germany

**Keywords:** EF hand, EH domain, Eps15 homology domain, molten globule, NMR spectroscopy, structure determination

## Abstract

Eps15 homology (EH) domains are universal interaction domains to establish networks of protein–protein interactions in the cell. These networks mainly coordinate cellular functions including endocytosis, actin remodeling, and other intracellular signaling pathways. They are well characterized in structural terms, except for the internal EH domain from human γ‐synergin (EHγ). Here, we complete the family of EH domain structures by determining the solution structure of the EHγ domain. The structural ensemble follows the canonical EH domain fold and the identified binding site is similar to other known EH domains. But EHγ differs significantly in the N‐ and C‐terminal regions. The N‐terminal α‐helix is shortened compared to known homologues, while the C‐terminal one is fully formed. A significant proportion of the remaining N‐ and C‐terminal regions are well structured, a feature not seen in other EH domains. Single mutations in both the N‐terminal and the C‐terminal structured extensions lead to the loss of the distinct three‐dimensional fold and turn EHγ into a molten globule like state. Therefore, we propose that the structural extensions in EHγ function as a clamp and are undoubtedly required to maintain its tertiary fold.

## Abstract 2

PDB Code(s): 2MX7;

## 1 INTRODUCTION

The Eps15 homology (EH) domain is an ~100 amino acid long interaction domain present in multiple proteins and conserved from fungi, plants, nematodes to mammals.^1^, ^2^, ^3^, ^4^ Three classes of peptides have been identified to interact with EH domains^5^, ^6^: Class I containing the NPF (asparagine‐proline‐phenylalanine)‐motif, class II containing the FW‐ (phenylalanine‐tryptophan), WW‐ (tryptophan‐tryptophan) or SWG‐ (serine‐tryptophan‐glycine) motifs and class III containing a H(S/T)F‐ (histidine‐serine/threonine‐phenylalanine) motif. The majority of EH domains bind the NPF‐motif of a given interaction partner,^5^, ^6^, ^7^ which is present in one or multiple copies. Multiple structures of EH domains have been determined by X‐ray crystallography and NMR spectroscopy over the years^8^, ^9^, ^10^, ^11^, ^12^, ^13^, ^14^, ^15^, ^16^ and they often have been successful targets of structural genomics consortia. They share the same overall fold, composed of two associated helix–loop helix motifs, known as EF hands, sometimes connected by a linker forming a short antiparallel ß‐sheet.^4^ EF hands are usually known for their Ca^2+^ binding properties, but not all EF hands possess all the residues required for Ca^2+^ binding, as defined by the canonical and pseudo‐EF hand consensus sequences.^17^, ^18^, ^19^, ^20^ If present, the bound calcium ion stabilizes the structural fold of the EH domain, but does not appear to play a role in calcium‐regulated events.^21^ In fact, some EH domains are completely devoid of calcium binding residues.^4^, ^21^

The NPF‐motif binds EH domains in a type I Asn‐Pro ß‐turn conformation and is almost completely buried in the binding pocket formed by a set of highly conserved residues of the second and third helix.^13^, ^14^, ^22^ The Phe residue of the NPF‐motif serves as a hydrophobic anchor point. Additional residues outside this motif can contribute to binding affinity and specificity in particular cases.^13^, ^14^, ^23^ An interaction between a single EH domain and this short recognition motif typically displays low affinity in binding (*K*_D_ value is in the high micromolar range) but the presence of multiple of these repeats increases binding affinity likely due to avidity effects.^6^, ^8^, ^22^, ^24^, ^25^ Moreover, the study of the Eps15‐Stonin2 complex revealed a novel mechanism by which high affinity and specificity between EH domains and their ligands can be achieved by the recognition of two NPF motifs by a single EH domain.^15^ Here, the first NPF‐motif binds to the canonical binding site on the EH domain, while the second motif inserts in a novel hydrophobic pocket on the backside of the molecule.

EH‐containing and EH‐interacting proteins are often implicated in the regulation of intracellular trafficking as well as cell signaling.^7^, ^21^, ^26^, ^27^, ^28^, ^29^ Known EH domain containing proteins in humans are Eps15, Eps15R, intersectin 1 and 2, Reps1, POB1, Eps15 Homology Domain protein (EHD)1–4 and γ‐synergin. Many of the listed proteins have been extensively studied in the past, but less is known about γ‐synergin.^29^, ^30^ This protein was identified through a yeast two‐hybrid screen with the large γ‐subunit of the AP‐complex, and a C‐terminal sequence stretch has been identified for binding of γ‐adaptin, which is found to be associated with clathrin at the trans‐Golgi network.^30^, ^31^ In another yeast two‐hybrid screen, the NPF repeat containing N‐terminal stretch (three NPF‐repeats followed by a coiled coil) of the secretory carrier membrane protein 1 (SCAMP1) was chosen to identify cytosolic adaptor proteins involved in endocytosis. γ‐synergin was found to be the major interaction partner and it has been proposed that the NPF‐repeats of SCAMP1 interact with the central EH domain of γ‐synergin.^32^

To date, from all the known human EH domain containing proteins, three‐dimensional structures have been determined by X‐ray crystallography or NMR spectroscopy for at least one of their EH domains with the exception of γ‐synergin. Here, we have been successful in determining the solution structure of the EH domain of γ‐synergin and analyzed its binding characteristics to NPF sequences derived from SCAMP1. We identified N‐ and C‐terminal structured extensions that are required to obtain a properly folded and functional interaction domain. We propose that these extensions act as a clamp needed to maintain the structural properties of both EF hands. Thus, replacing a single proline to an alanine residue comprising either the N‐ or C‐terminal extension is sufficient to turn the properly folded EH domain into a molten globule state. Our work completes the family of human EH domain structures including dynamic and functional insights into NPF binding. It expands for this reason the general knowledge on the EH domain fold.

## 2 RESULTS AND DISCUSSION

### 2.1 Construct design and SCAMP1 interaction

**FIGURE 1:**
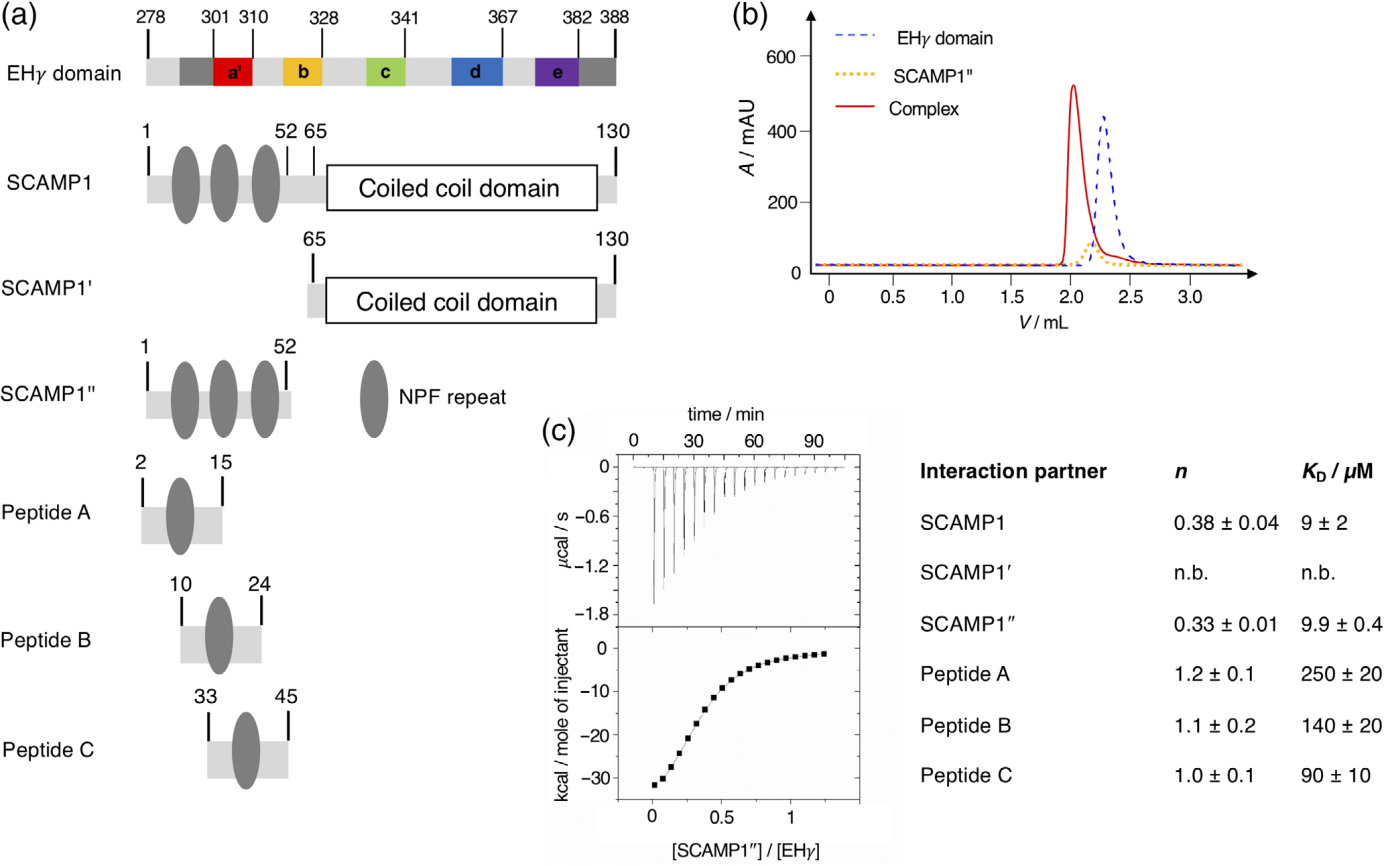
Composition of the EH𝛾 domain and initial screen of potential interaction to different SCAMP1 constructs. (a) Primary outline of the EH𝛾 domain comprising structural elements a′, b, c, d, and e determined as α‐helical in this study as well as structured N‐ and C‐terminal extensions that are highlighted in dark‐gray (see also Figure 2). The N‐terminal SCAMP1 construct is devoid of any transmembrane helices and comprises two domains that can be divided into a coiled coil (SCAMP1′) and NPF repeat rich motifs (SCAMP1″). SCAMP1″ has been further subdivided into peptide A, B and C in this study enabling to probe individual interactions to the EH𝛾 domain. The primary sequences of all molecules probed in this study are shown in SI Table 1. (b) Probing the interaction between the EH𝛾 domain and SCAMP1″ applying analytical size exclusion chromatography. The formed complex (continuous line, colored in red) eluates at a lower retention volume compared to the isolated EH𝛾 domain (dashed line, colored in blue) and SCAMP″ (dotted line, colored in orange). (c) Following the interaction between the EH𝛾 domain and SCAMP1″ using isothermal titration calorimetry (ITC). The results of the quantitative analyses applying a single site binding model are presented right to the ITC profile, n.b. refers to no binding

Based on available X‐ray and NMR structures of homologous EH domains we initially designed an expression construct of the EH domain of γ‐synergin (EHγ) for structural, dynamic, and functional characterization. The EH domain fold is highly conserved and known structures often display an unstructured N‐terminus, followed by four helices organized as two EF hands, and a short C‐terminal 3_10_ helix or an unstructured C‐terminus.^4^ Our first construct of EHγ (residues 295–388) covered the known EH domain fold, but was poorly expressed, highly prone to aggregation and did not bind a respective NPF repeat. We extended the N‐terminal region by four (residues 291–388) and 16 residues (residues 279–388, Figure 1a and SI Figure 1). Although the N‐terminal extensions were outside the conserved EH domain fold based on the sequence alignment of human EH domains, the longer constructs showed enhanced expression characteristics. Since the longest EHγ construct was expressed in soluble form and could be purified to high purity and yields, we proceeded with this construct for further studies. From here on, EHγ refers to the longest construct covering residues 279–388. EHγ bound the respective NPF‐repeats from the N‐terminal domain of SCAMP1 (Figure 1a and SI Table 1) with micromolar affinity as evident by isothermal titration calorimetry (Figure 1c and SI Figure 2). This interaction was further confirmed by monitoring complex formation via analytical size exclusion chromatography where both proteins eluted as a complex at lower retention times (Figure 1b). As expected, the interaction between individual NPF–repeat constructs (peptides A–C) and EHγ was weaker, while a SCAMP1 construct devoid of NPF‐repeats did not bind to EHγ at all. This indicates that the N‐terminal NPF‐repeats of SCAMP1 are solely responsible for the interaction with EHγ (Figure 1). In summary, we have generated a construct of EHγ (residues 279–388) that can be expressed and purified at high yields, binds its interaction partner SCAMP1 and is therefore well suited for in depth structural and dynamical studies.

### 2.2 Solution structure of EHγ

**FIGURE 2:**
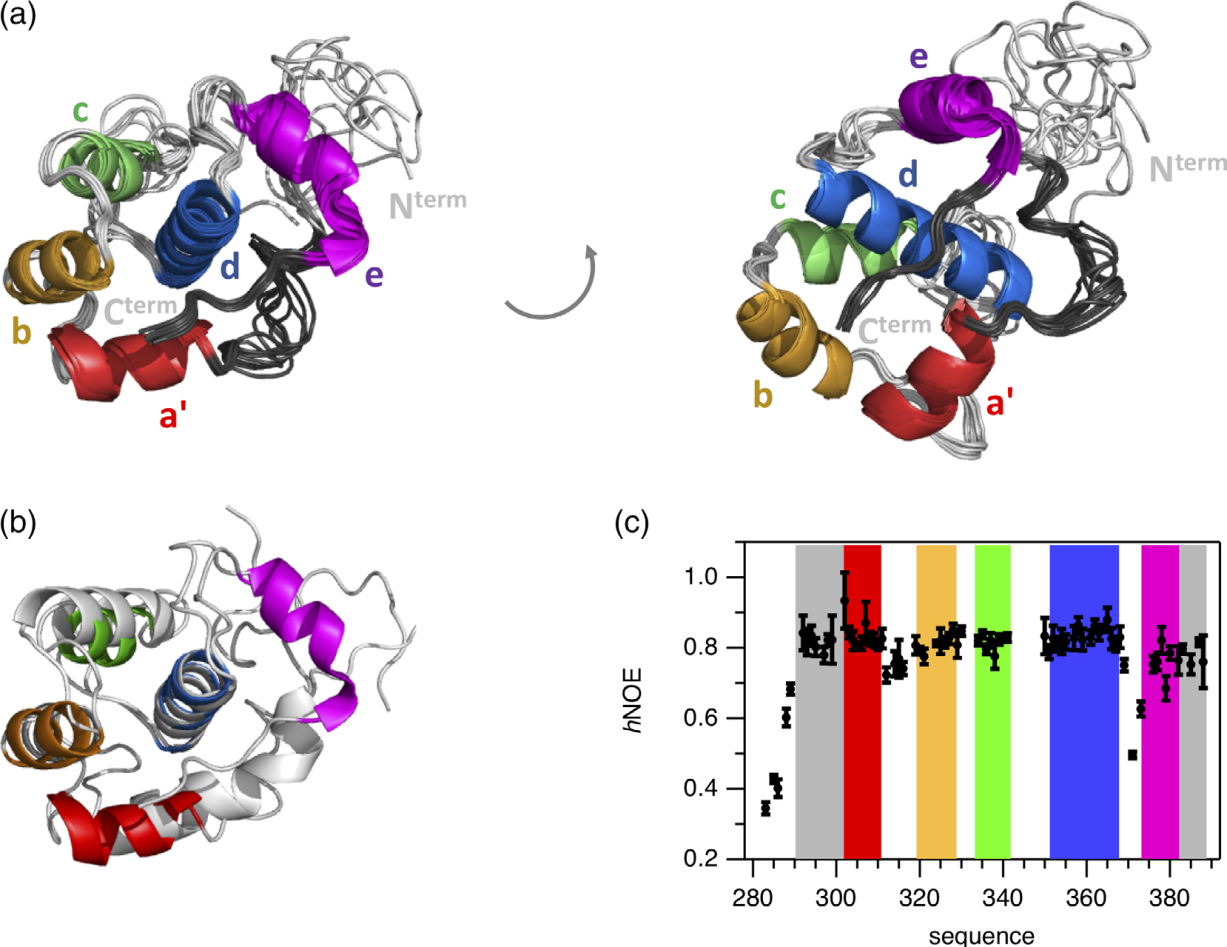
Structural and dynamical characterization of the EH𝛾 domain. (a) The three‐dimensional structure of the EH𝛾 domain is shown as an alignment of the 10 lowest energy structures. α‐Helices are color‐coded and labeled. Also, well‐structured regions N‐terminal of α‐helix a′ and C‐terminal of helix e have been colored (dark‐gray). Accompanying SI Table 3 lists NMR restraints and statistical analysis of the NMR‐based structure calculation. (b) Comparison between the lowest energy structure of EH𝛾 determined in this study (helical elements highlighted in color) to homologues EH domain of EHD1 possessing pdb code 2JQ6 (colored in light grey). The alignment has been conducted for backbone atoms comprising helix d (K352‐R367 in EH𝛾 and D103‐E118 in EHD1). Further individual comparisons between EH𝛾 and homologues structures are shown in SI Figure 4. (c) {^1^H‐}^15^N heteronuclear NOE ( *h* NOE) acquired for the EH𝛾 domain at *T* = 298 K and *B*_0_ = 20 T reporting on backbone dynamics on the pico‐to‐nanosecond time scale. Colors used for highlighting the background refer to the structural composition presented in panel a. Error bars refer to the standard deviation obtained from three independent measurements

Since the various EHγ constructs resisted crystallization we determined its three‐dimensional structure by solution state NMR spectroscopy following established protocols. The two‐dimensional ^1^H‐^15^N‐HSQC spectrum of EHγ is well dispersed and thus 75 cross‐peaks representing backbone resonances could be unambiguously assigned (SI Figure 3). Since the EHγ construct chosen for structure determination contains 15 proline residues, we investigated their *cis*/*trans* conformations under equilibrium conditions. In total, 13 out of 15 prolines were identified to be in *trans* conformation derived by ^13^C_β_,^13^C_γ_ chemical shifts and/or NOEs information (SI Table 2). Even though for the two remaining proline residues (P290 and P347) the conformation could not be directly determined because of missing spectral information, they likely adopt a *trans* conformation as well. Thus, we determined an *all trans* three‐dimensional structure of EHγ using out of 1,170 NOEs, 185 TALOS derived phi/psi dihedral angles and 44 residual dipolar couplings (RDCs) restraints (Figure 2a and SI Table 3). The structures possessing lowest energy highlight that EHγ consists of five helices (a′, b, c, d, e), forming two EF hand motifs (helices a′ and b and helices c and d, respectively). The N‐terminal helix a′ is shortened compared to other EH domain structures (therefore named a′) and the C‐terminal helix e represents a fully formed α‐helix instead of a short 3_10_ helix observed in other homologues (Figure 2b and SI Figure 4). In addition, regions that are N‐terminal of helix a′ (residues 290–300) and C‐terminal of helix e (residues 380–388) are also well structured. The somewhat lower order of residues comprising the N‐terminal structured region (compared to residues forming the C‐terminal extension) is likely caused by a lower number of structural restraints that have been used for structure calculation. The rigid nature of all five helices and both structural extensions was independently verified by an {^1^H‐}^15^N heteronuclear NOE experiment, which senses backbone motions on the pico‐to‐nanosecond time scale. Values above 0.7 indicate high structural order (Figure 2c). In contrast, the N‐terminus up to residue 289 displays increased dynamics and is likely disordered as seen by the poor alignment in the structural ensemble (Figure 2a) and heteronuclear NOE values below 0.7 (Figure 2c). The loop connecting helices d and e displays higher backbone dynamics in the heteronuclear NOE experiment, but is well defined in the structural ensemble. There is no evidence that the two EF hands bind Ca^2+^, since they lack classical Ca^2+^ binding side chains.

Overall, EHγ displays the canonical EH domain fold but differs in certain aspects to homologues structures: (i) presence of a shortened helix a′, (ii) presence of an additional helix e, and (iii) well‐structured extensions at both N‐ and C‐terminal regions.

### 2.3 Binding of EHγ to ΝPF‐repeats from SCAMP1

**FIGURE 3:**
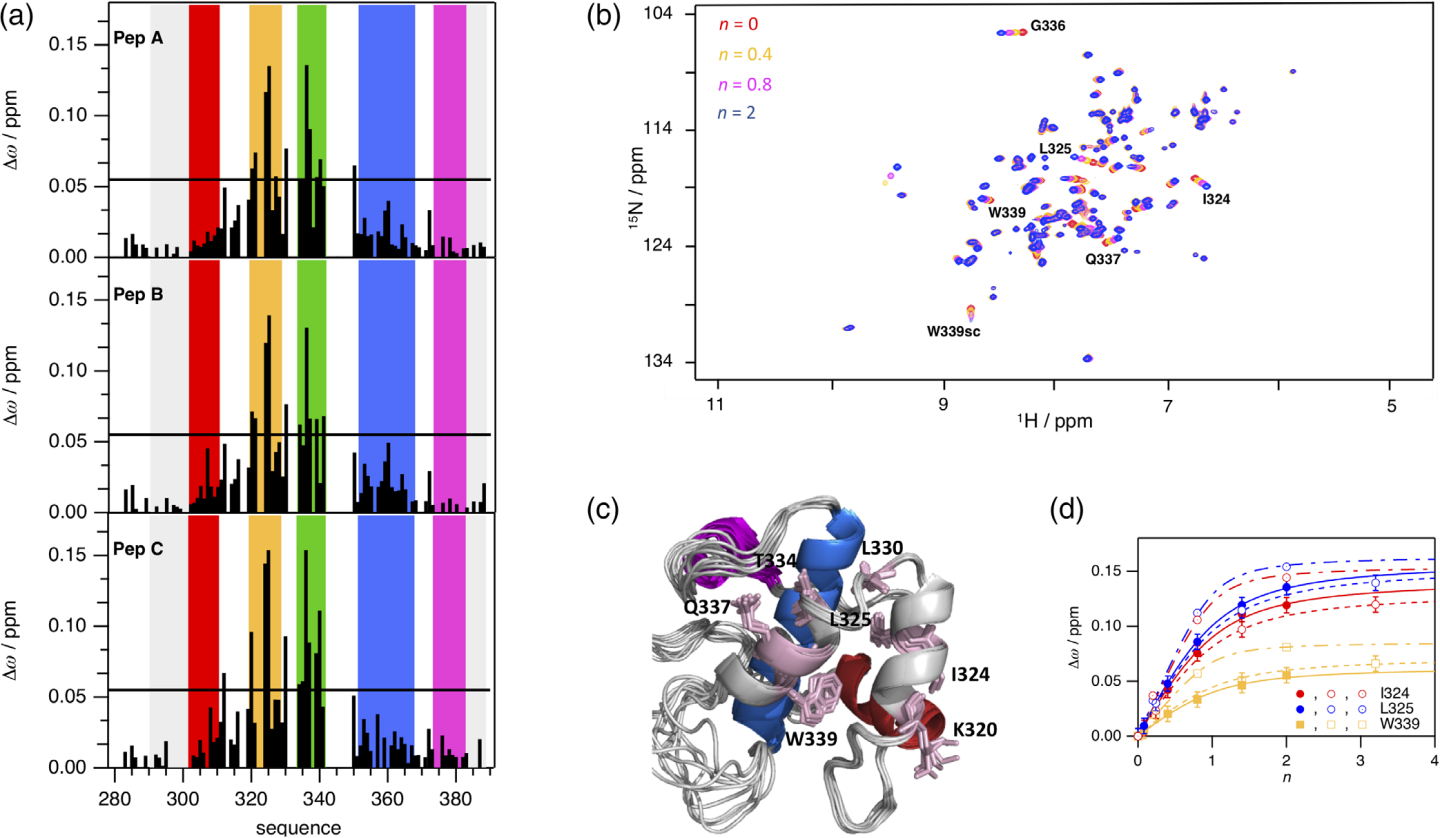
Interaction between the EH𝛾 domain and peptides comprising the NPF motif found in SCAMP1. (a) Analysis of changes of chemical shifts of NMR resonance signals following the interaction between isotopically labeled EH𝛾 and peptide A (top), B (mid), and C (bottom). Colors used for highlighting the background refer to the structural composition of EH𝛾 presented in Figure 2a. Values for Δ𝜔 have been calculated using the molar ratio *n* = 2 between peptide and EH𝛾, respectively. The cutoff value identifying most affected residues has been set for all titration experiments to Δ𝜔 = 0.055 ppm. (b) Overlay of two‐dimensional heteronuclear ^1^H‐^15^N HSQC NMR spectra following the interaction between peptide A and EH𝛾 acquired for different stoichiometric ratios at *T* = 298 K and *B*_0_ = 20 T: *n* = 0 (colored in red), *n* = 0.4 (colored in orange), *n* = 0.8 (colored in magenta), *n* = 2 (colored in blue). Residues of EH𝛾 that are most affected upon binding are labeled by using the one letter code for amino acids followed by the position in the primary sequence, sc refers to side chain. (c) Highlighting all residues of EH𝛾 exceeding Δ𝜔 = 0.055 ppm in all three titration experiments shown in panel A. These residues are presented including side chains (stick mode) in pink and labeled. (d) Individual titration profiles observed for ^1^H‐^15^N correlations of I324 (colored in red), L325 (colored in blue), and W339 (colored in orange) of EH𝛾 when peptide A (closed symbols), peptide B (open symbols), or peptide C (symbols with inner dot) has been stepwise added. The binding affinity has been determined to *K*_D_^pepA^ = 110 ± 10 μM (continuous line), *K*_D_^pepB^ = 120 ± 30 μM (dashed line), *K*_D_^pepC^ = 30 ± 20 μM (dot‐dash line) by applying a joint fitting procedure for these residues to an one site binding model

To map the binding site of NPF repeats from SCAMP1 on EHγ, we performed three independent NMR spectroscopically detected binding experiments, titrating ^15^N‐labeled EHγ with unlabeled SCAMP1 derived peptides named A, B, and C (Figures 1 and 3), each containing a single NPF‐repeat. Individual NMR cross‐peaks changed their chemical shift values with increasing peptide concentrations as monitored in two‐dimensional ^1^H‐^15^N HSQC spectra, indicative for the fast NMR exchange limit and short lifetimes of the complexes. The signals that shifted the most are located on helices b and c in agreement to observations of homologous EH domains (Figure 3a,b). Mapping the changes of chemical shifts on the three‐dimensional structure of EHγ illustrates that they cover the cleft between helices b and c, comprising the highly conserved Trp339 as potential key residue for NPF‐repeat binding (Figure 3c). The NPF binding site is distant to the secondary structure elements that are different in EHγ compared to other EH domains (see above). Therefore, we expect a similar binding mode compared to other known EH domain‐NPF structures.^13^, ^14^, ^22^ We quantified the binding interaction of all three peptides to EHγ by a global analysis of the residues with the largest changes in chemical shifts (Figure 3d) and determined respective *K*_D_ values. While peptides A and B bind with an affinity of around 100 μM, the affinity of peptide C is about 30 μM. This higher affinity of peptide C indicates potential additional interactions to EHγ next to the NPF‐motif. In summary, the binding site of EHγ to NPF‐motif containing peptides could be mapped to a single common binding site in the cleft between helices b and c, which is similar to other EH domains and distant from the regions of structural differences. A closer look to the ITC derived enthalpic and entropic contributions to binding of different SCAMP constructs and peptides further extends this picture (SI Figure 2). There is no enthalpic gain in EHγ binding to SCAMP1″ (possessing three NPF‐motifs) compared to the sum of binding of peptides A, B, and C that would indicate interactions to different EHγ domains. In fact, enthalpic gains are somewhat reduced in SCAMP1″. The higher binding affinity of SCAMP1″ arises purely from a largely reduced entropic penalty, which origins from covalent linking of the three NPF‐motifs.

### 2.4 N‐ and C‐terminal extensions form a clamp in EHγ

**FIGURE 4:**
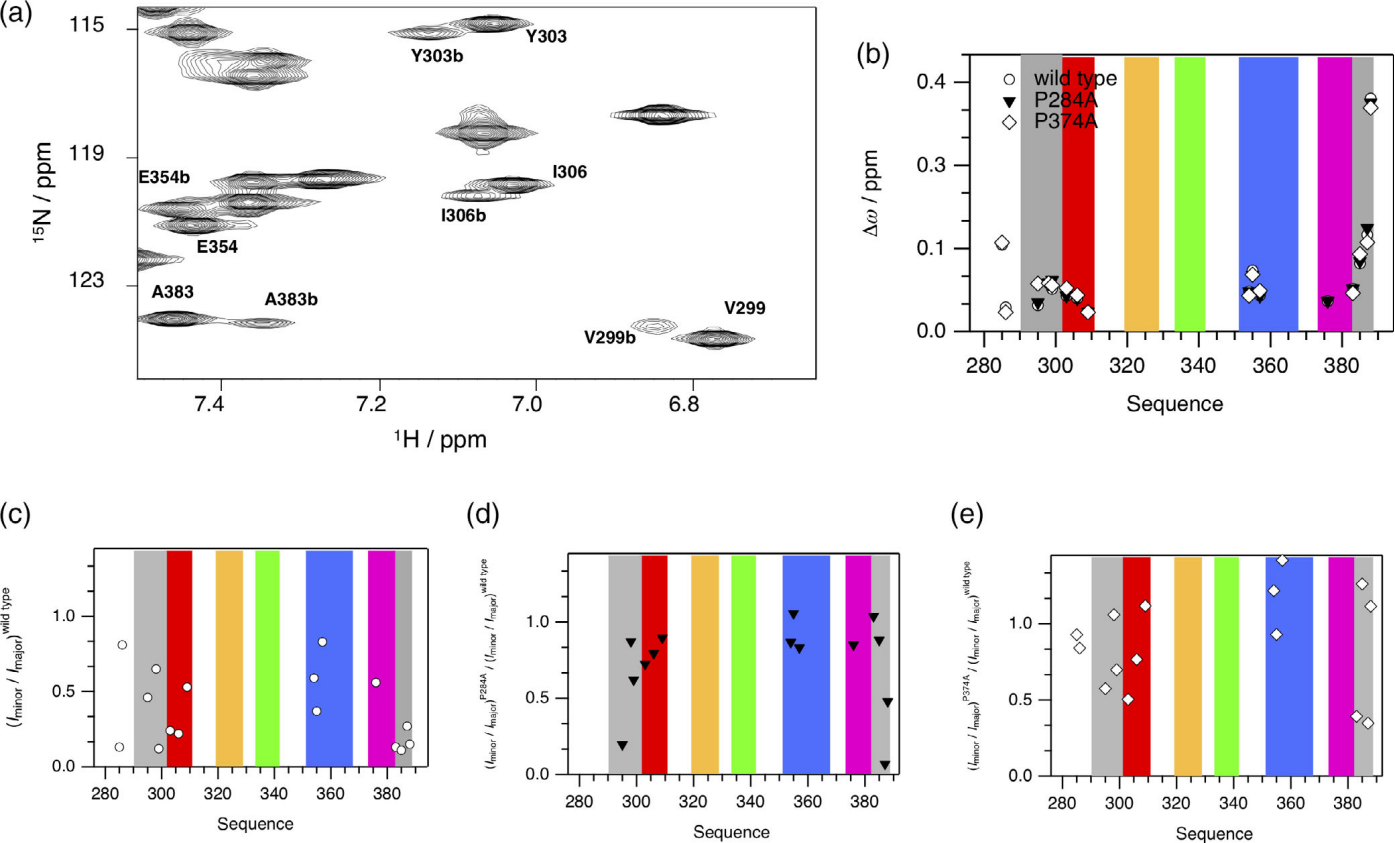
Conformational heterogeneity of EH𝛾. (a) Section of two‐dimensional heteronuclear ^1^H‐^15^N HSQC NMR spectrum acquired for EH𝛾 reveals at least two conformations that possess different chemical shifts for a distinct set of residues (cross‐peaks arising from the minor conformation are indicated by “b” following the position in the primary sequence). (b–e) Characterization of two conformations observed for EH𝛾 and two variants P284A and P374A regarding differences in chemical shifts (b) and in the ratio of signal heights (c–e) observed in an ^1^H‐^15^N‐HSQC NMR spectrum. Colors used for highlighting the background refer to the structural composition of EH𝛾 presented in Figure 2a. Please note that no spectroscopic information could be obtained for A285 and Q286 (P284A variant) as for A376 (P374A variant)

EHγ structurally differs from known EH domains by an N‐terminally shortened helix a′, which is replaced by an N‐terminally structured extension (residues 290–300), and by an additional helix e in conjunction with a C‐terminally structured extension (residues 380–388). Both extensions primarily interact with helix d, but also directly with each other via Trp 292. These regions display structural heterogeneity as evident by additional cross‐peaks in the ^1^H‐^15^N‐HSQC spectrum that overall possess lower signal heights compared to the remaining cross‐peaks (Figure 4a and SI Figure 3). The structural ensemble illustrated here, represents the major conformation of EHγ. We argue that the additional conformation or conformations are structurally similar to EHγ, because the additional cross‐peaks are very close to the cross‐peaks observed for the main conformation, indicating a similar structural environment (Figure 4a,b). Moreover, their {^1^H‐}^15^N heteronuclear NOE values are also high (SI Figure 5), confirming the well‐structured nature of the additional conformations.

Due to the high number of proline residues in the EHγ construct (15 residues in total), we hypothesized that potential proline *cis*/*trans* conformations are the reason for the structural heterogeneity. Therefore, we created EHγ variants P284A, P290A, P374A, and P386A. The chosen mutation sites are located in the unstructured N‐terminal region (P284A), at the beginning of the N‐terminal structured extension (P290A), in the turn connecting helices d and e (P374A) and at the end of the C‐terminal structural extension (P386A), respectively. The two‐dimensional ^1^H‐^15^N HSQC NMR spectra of the variants P284A and P374A show a disappearance of the additional cross‐peaks representing residues that are in close proximity to the mutation site (Figure 4c–e). Therefore, we conclude that the structural heterogeneity observed in EHγ likely originates from the presence of multiple minor conformations caused by proline *cis*/*trans* equilibria.

**FIGURE 5:**
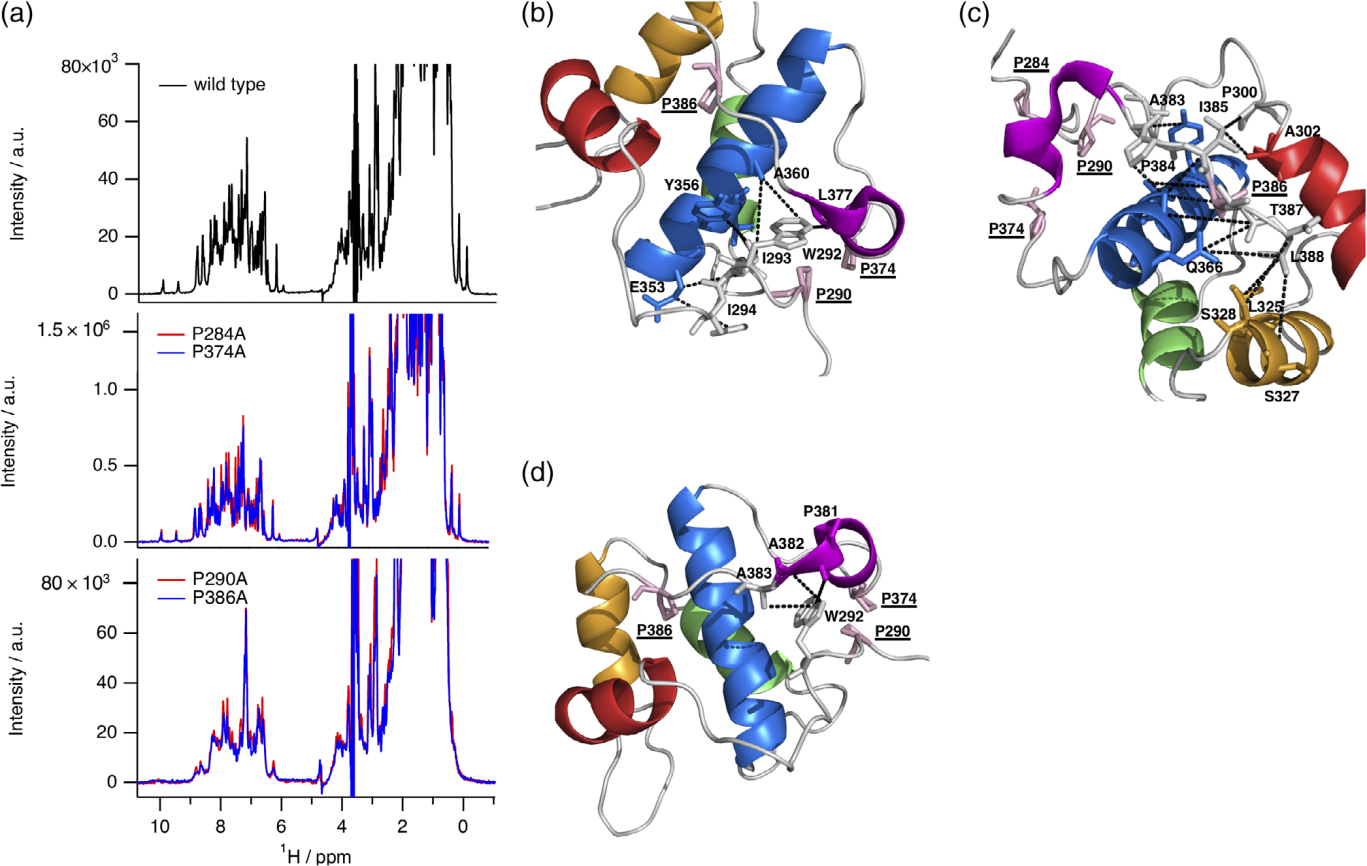
Structural impact of selected proline residues comprising EH𝛾. (a) Overlay of one‐dimensional proton NMR spectra acquired for wild type (top), P284A (colored in red) and P374A (colored in blue) (mid) as well as P290A (colored in red) and P386A (colored in blue) protein variants (bottom). (b) Network of NOEs illustrating structural contacts between residues comprising helices d (colored in blue), e (colored in magenta) and N‐terminal residues. (c) Network of NOEs illustrating structural contacts between residues comprising helices a’ (colored in red), b (colored in orange), d (colored in blue) and C‐terminal residues. (d) Network of NOEs illustrating structural contacts between N‐ and C‐terminal residues. Proline residues used for particular alanine replacement are shown with side chains (stick mode, colored in pink) and have been underlined. NOE contacts have been highlighted by dotted lines (colored in black)

In case of the P290A and P386A variants we observed drastic changes in the one‐dimensional ^1^H NMR spectra compared to wild type and both other proline mutation sites (P284A and P374A) (Figure 5a), with characteristics of a molten globule state.^33^ These spectra display much broader lines with a certain degree of dispersion, but lack upfield and downfield shifted resonance signals, that are indicative of a three‐dimensional structure. It appears that in the absence of P290 or P386 the local restriction of backbone conformations is reduced, resulting in a loss of the distinct three‐dimensional structure of EHγ while individual secondary structures (helices) or an undefined ensemble of multiple tertiary conformations remain. This explanation is further supported by independent biochemical and biophysical data. Thus, molten globule state like variants P290A and P386A elute at lower retention volumes on gel filtration compared to the wild type protein. They are also highly prone to proteolytic degradation by proteases and display reduced secondary structural content as evident by circular dichroism spectroscopic data (SI Figure 6). P290 is located at the beginning of the N‐terminal structured extension next to Trp292 and Ile 293, which mediate key long‐range NOE contacts to the C‐terminally structured extension and helix d (Figure 5b,d). P386 is localized at the end of the C‐terminally structured extension with crucial interactions to helices a′, b, and d (Figure 5c). If these interaction networks are significantly perturbed due to mutation (e.g., P290A or P386A), the well‐defined tertiary structure of EHγ is lost, highlighting the importance of both N‐ and C‐terminally structured extensions for overall stability and structural integrity. This is also depicted by our initial observation that designed constructs lacking the N‐terminal extension are poorly expressed, highly prone to aggregation and likely not properly folded. Taken together, we propose that the structural extensions in EHγ function as a clamp and are undoubtedly required for the tertiary fold of EHγ. The extensions might present an alternative way to stabilize the EHγ fold.

## 3 CONCLUSIONS

Multiple studies have addressed structure determination of EH domains in the past, with a particular emphasis on N‐terminal or C‐terminal EH domains. Due to their small size, these domains were also targets for structural genomics consortia applying NMR‐spectroscopy or X‐ray crystallography. Numerous structures of EH domains from human proteins are accessible in the Protein Data Bank except for γ‐synergin. Our work presented here, completes the family of human EH domains. EHγ is a bona fide member of the EH family and binds NPF‐repeat substrates via its conserved hydrophobic pocket as illustrated for other EH domains. The reason why EHγ has resisted structure determination until recently is likely due to its requirement of additional secondary structure elements outside the core EF hand helices which act as a clamp to keep the three‐dimensional structure intact. This highlights that sequence stretches outside the structural fold can have crucial contributions in folding and maintaining the structure. Furthermore, the sequence of EHγ is highly enriched in proline residues causing a certain degree of structural heterogeneity under native conditions. Whether this has any functional implications needs to be shown in future work.

## 4 MATERIALS AND METHODS

### 4.1 Reagents

Isopropyl‐β‐d‐thiogalactopyranoside (IPTG) was purchased from Anatrace (Maumee, OH, USA). Lysogeny broth medium was from Becton Dickinson (Franklin Lakes, NJ, USA) and terrific broth was from Formedium (Norfolk, UK). The peptides A, B, and C used in this study had amidated C‐termini and were purchased from GL Biochem Ltd (Shanghai, China). The sequences of all used peptides are given in SI Table 1. All other chemicals were of analytical grade and obtained from Sigma‐Aldrich, unless otherwise stated.

### 4.2 Gene construction

The gene coding for γ‐synergin has been purchased from Source Bioscience (IMAGp998O0612737Q). The DNA for the different EHγ constructs was amplified by PCR and cloned into the pNIC28‐Bsa4 vector using ligation independent cloning.^34^, ^35^, ^36^ This vector possesses an N‐terminal histidine‐tag followed by a TEV cleavage site, resulting in two additional residues (Serine‐Methionine) at the N‐terminus after tag‐cleavage. The same cloning procedure was used for the soluble SCAMP1 fragments (residues 1–130, 1–52, and residues 65–130). The SCAMP1 gene was obtained from the hORFeome collection (http://horfdb.dfci.harvard.edu). All vectors possess a T7 promoter and terminator sequence. Single point mutations were introduced by blunt‐end PCR.

### 4.3 Protein expression and purification

Genes coding for the different EHγ and SCAMP constructs were transformed into *Escherichia coli* BL21(DE3) expression cells (Novagen) and soluble expression was induced at either 20°C with 0.2 mM IPTG for 18 h or at 37°C with 1 mM IPTG for 4 h. Initial soluble over‐expression screening after cell lysis via sonication was monitored by SDS‐PAGE. For large scale production of native unlabeled protein, cells were typically grown in TB medium and induced with 1 mM IPTG at 37°C for 4 h. The cell pellets were resuspended in lysis buffer (20 mM Tris (pH 7.5), 300 mM NaCl, 5% glycerol, 15 mM imidazole, 2 mM CaCl_2_, 5 units/ml DNase I, 1 tablet of protease inhibitors (Roche) per 100 ml buffer, 1 mg/ml lysozyme, 0.5 mM TCEP) and the bacteria were lysed by three passages through an emulsifier (EmulsiFlex‐C3, Avestin) with a maximum pressure of 10,000 psi. The lysate was centrifuged (20 min, 19,000*g*) and incubated with 2 ml of Ni‐IMAC beads (ThermoFisher) per 1 L of culture on a rotatory wheel. The lysate was then transferred into a gravity column and washed twice with 10 ml wash buffer (20 mM Tris (pH 7.5), 300 mM NaCl, 5% glycerol, 15 mM imidazole, 2 mM CaCl_2_, 0.5 mM TCEP). The bound protein was eluted with 10 ml and subsequently with 5 ml of elution buffer (20 mM Tris (pH 7.5), 150 mM NaCl, 5% glycerol, 250 mM imidazole, 2 mM CaCl_2_, 0.5 mM TCEP). The elution fractions were pooled and 0.5 mg of TEV protease per liter of bacterial culture was added. The samples were dialyzed (2 kDa cutoff) against 500 ml wash buffer overnight. Next day, the samples were incubated on a gravity column with 1 ml Ni‐beads per 1 L of culture. The flow‐through was concentrated (5 kDa cutoff) to maximum of 10 mg/ml and further purified by size exclusion chromatography on a Superdex 75 HiLoad column using gel filtration buffer (20 mM Tris (pH 7.5), 100 mM NaCl, 2 mM CaCl_2_). Finally, the samples were concentrated (5 kDa cutoff concentrator) up to 20 mg/ml and either directly used or flash‐frozen for later use. All steps were performed at 4°C. ^15^N and ^15^N/^13^C isotope‐labeled NMR samples were produced using M9 minimal media based on ^15^NH_4_Cl and ^13^C‐Glucose as nitrogen and carbon source (Spectra Stable Isotopes, USA) and supplemented with vitamin mixture. Purification of all constructs and mutants was performed as essentially described above.

### 4.4 Isothermal titration calorimetry

ITC measurements were performed on a VP‐ITC instruments (GE Healthcare, Chalfont St. Giles, UK). The calorimetric cell (with a total cell volume of 1,400 μl) contained 50–200 μM EHγ in 20 mM Tris, 100 mM NaCl, 2 mM CaCl_2_ pH 7.5. SCAMP1 fragments and peptides A, B, C at 700–2000 μM were titrated into the cell at 20°C. The heat generated after each ligand injection was obtained by integration of the calorimetric signal. Resulting binding isotherms were analyzed according to an one site binding model using the Origin software (OriginLab Corp., Northampton, MA, USA).

### 4.5 Limited proteolysis

Stability tests for the expressed EHγ mutants against proteolytic degradation was performed in gel filtration buffer at room temperature in the presence of chymotrypsin (chymotrypsin to target protein ratio: 1∶5,000). The reaction was stopped after different time points by addition of SDS sample buffer and subsequent heating. The samples were then analyzed on SDS‐PAGE.

### 4.6 Analytical gel filtration

The quality and oligomeric state of the EHγ construct was assessed on an analytical gel filtration column (Superdex 75 5/150 GL, GE Healthcare). To guarantee reproducible and reliable gel filtration runs, an ÄKTAmicro™ (GE Healthcare) was coupled to an auto sampler, which automatically injected with high‐precision 25 μl of protein sample.^36^ Analytical gel filtration runs were performed in duplicates in the cold room at a flow rate of 0.2 ml/min in gel filtration buffer (20 mM Tris pH 7.5, 100 mM NaCl, 2 mM CaCl_2_). The used EHγ construct was injected spanning a concentration range of 10–1,000 μM. The column has been calibrated with protein standards ranging from 6.5 to 75 kDa. Interactions between EHγ and the SCAMP 1–52 fragment were also probed using this analytical gel filtration setup; the proteins alone or the potential complex (at concentrations of 100 μM EHγ and/or 100 μM SCAMP 1–52) were applied to a Superdex™ 200 5/150 GL analytical gel filtration column and analyzed. Analytical gel filtration runs were performed at 4°C at a flow rate of 0.2 ml/min in the gel filtration buffer.

### 4.7 Equilibrium CD spectroscopy

Far‐UV‐CD spectra on EHγ wild type and mutants were recorded at 20°C in gel filtration buffer with a JASCO J600A spectropolarimeter (0.1 cm cell length, 20 μM protein concentration, 1 nm bandwidth) and corrected for the buffer contributions. Corrected CD spectra were analyzed with the online software package Dichroweb.^37^

### 4.8 NMR spectroscopy

NMR experiments were conducted on a Bruker Avance III 600 spectrometer (assignment and determination of NOEs, performing titration experiments) and on a Bruker Avance III HD 850 spectrometer (determination of {^1^H‐}^15^N heteronuclear NOE and RDCs, probing PxA variants of EHγ), all at *T* = 298 K. Sample concentration was around 1 mM EHγ in 20 mM Tris pH 7.5, 100 mM NaCl, 2 mM CaCl_2_, 10% (vol/vol) D_2_O. Initial experiments showed that the NMR spectra are identical at concentrations of EHγ between 50 μM and 1 mM. Spectra were processed using NMRPipe^38^ and analyzed using NMRView.^39^ For RDC measurements, EHγ was aligned in pf1 phages and ^1^H‐^15^N HSQC NMR spectra have been acquired using in‐ and anti‐phase mode in ^15^N dimension. For titration experiments, stock solutions of unlabeled peptide have been stepwise added to ^15^N labeled EHγ achieving stoichiometric excess of *n* = 2 (Pep A, C) and *n* = 3.2 (Pep B) to obtain saturation of binding. The {^1^H}‐^15^N NOE experiments were performed using a train of 120° pulses for 3 s, the inter‐scan relaxation period was also set to 3 s.

### 4.9 Structure calculation

In total, 1,078 chemical shifts have been assigned in ^1^H, ^13^C, and ^15^N dimension. No chemical shift information could be obtained for four residues comprising the loop between helix c and helix d (A342, T346, P347, K349).

Backbone resonances were assigned using HNCA, HNCACB, and HN(CO)CACB experiments. Side chain information was obtained via H(C)CH‐TOCSY and NOEs. NOEs for the structure determination were derived from 3D NOESY‐edited HSQC experiments for ^15^N and ^13^C aliphatic/aromatic nuclei, which were also used to confirm and finalize the side chain assignment. Phi‐Psi dihedral angle constraints were derived using TALOS+.^40^ Alignment tensors were calculated using Tensor.^41^ Structures were calculated using ARIA2.1^42^ with standard parameters. A correlation plot between experimentally determined and back‐calculated RDC values is shown in SI Figure 7.

### 4.10 Protein structure accession number

The coordinates of the structure of the EHγ domain have been deposited in the Protein Data Bank under the accession number 2MX7. The assignment of chemical shift values has been deposited in the BMRB under the accession number 25395.

## CONFLICT OF INTEREST

Authors declare that they have no competing interests.

## AUTHOR CONTRIBUTIONS

**Michael Kovermann:** Conceptualization (equal); data curation (equal); formal analysis (equal); funding acquisition (equal); investigation (equal); project administration (equal); resources (equal); supervision (equal); validation (equal); visualization (equal); writing – original draft (equal); writing – review and editing (equal). **Ulrich Weininger:** Conceptualization (equal); data curation (equal); formal analysis (equal); funding acquisition (equal); investigation (equal); project administration (equal); resources (equal); supervision (equal); validation (equal); visualization (equal); writing – original draft (equal); writing – review and editing (equal). **Christian Low:** Conceptualization (equal); data curation (equal); formal analysis (equal); funding acquisition (equal); investigation (equal); project administration (equal); resources (equal); supervision (equal); validation (equal); visualization (equal); writing – original draft (equal); writing – review and editing (equal).

## Supporting information


**Data S1**: Supporting Information.
