# Supplementary material for: Completing the family of human Eps15 homology domains: Solution structure of the internal Eps15 homology domain of γ‐synergin
Source: Protein Sci. 2022 Jan 12;31(4):811–21. doi: 10.1002/pro.4269 (PMC8927860; doi:10.1002/pro.4269)
Supplement: Supplementary file 1 — Data S1: Supporting Information. [file PRO-31--s001.pdf]

Supplementary Materials

Completing the family of human EH domains: Solution structure of the internal EH domain of γ-synergin

Michael Kovermann[1,2*], Ulrich Weininger[3*] and Christian Löw[4*]

[1] Department of Chemistry, University of Konstanz, Universitätsstrasse 10, 78457 Konstanz, Germany

[2] Konstanz Research School Chemical Biology KoRS-CB, University of Konstanz, Universitätsstrasse 10, 78457 Konstanz, Germany

[3] Institute of Physics, Biophysics, Martin-Luther-University Halle-Wittenberg, D-06120 Halle (Saale), Germany

[4] Centre for Structural Systems Biology (CSSB), Notkestrasse 85, D-22607 Hamburg, Germany Molecular Biology Laboratory (EMBL), Hamburg Unit c/o Deutsches Elektronen Synchrotron (DESY), Notkestrasse 85, D-22607 Hamburg, Germany

*Corresponding authors:

Michael Kovermann

University of Konstanz, Universitätsstrasse 10, 78457 Konstanz, Germany.

phone: +49 7531 88 3801

email: michael.kovermann@uni-konstanz.de

Ulrich Weininger

Martin-Luther-University Halle-Wittenberg, D-06120 Halle (Saale), Germany.

phone: +49 345 55 28555

email: ulrich.weininger@physik.uni-halle.de

Christian Löw

European Molecular Biology Laboratory Hamburg, Notkestrasse 85, D-22607 Hamburg, Germany.

phone: +49 40 8998 87570

e-mail: christian.loew@embl-hamburg.de

ORCIDs:

https://orcid.org/0000-0002-3357-9843 Michael Kovermann

https://orcid.org/0000-0003-0841-8332 Ulrich Weininger

https://orcid.org/0000-0003-0764-7483 Christian Löw

SI Table 1: Constructs and sequences

**Sequence of EHγ:**<br>Construct for structure determination: construct 279-388 with 2 additional residues from the plasmid<br>SMFPSQDPAQPRMPPWIYNESLVPDAYKKILETTMTPTGIDTAKLYPILMSSGLPRETLGQIWALANR<br>TTPGKLTKEELYTVLAMIAVTQRGVPAMSPDALNQFPAAPIPTL
**SCAMP1 (1-130):**<br>SMMSDFDSNPFADPDLNNPFKDPSVTQVTRNVPPGLDEYNPFSDSRTPPPGGVKMPNVPNTQ<br>PAIMKPTEEHPAYTQIAKEHALAQAELLKRQEELERKAAELDRREREMQNLSQHGRKNNW<br>PPLPSNFPVG
**SCAMP1' (65-130):**<br>SMKPTEEHPAYTQIAKEHALAQAELLKRQEELERKAAELDRREREMQNLSQHGRKNNW<br>PPLPSNFPVG
**SCAMP1'' (1-52):**<br>SMMSDFDSNPFADPDLNNPFKDPSVTQVTRNVPPGLDEYNPFSDSRTPPPGGVK
**Peptide A (Y added at the C-term for concentration determination):**<br>NH_2-SDFDSNPFADPDLNY-$CONH_2$
**Peptide B (Y added at the C-term for concentration determination):**<br>NH_2-ADPDLNNPFKDPSVTY-$CONH_2$
**Peptide C:**<br>NH_2-GLDEYNPFSDSRT-$CONH_2$

SI Table 2: Main conformation of proline residues in EHγ

	$^{13}C\beta$/ ppm	$^{13}C\delta$/ ppm	Δ ($^{13}C\beta$-$^{13}C\delta$) / ppm	NOE to (i-1)[a]
trans Pro	**32.22**[a]	**27.32**[a]	**4.90**	δ
cis Pro	**34.80**[a]	**24.90**[a]	**9.90**	α
Pro 280	31.90	27.22	4.68	
Pro 284	32.03	n. d.		
Pro 287	31.90	27.22	4.68	δ
Pro 290	n. d.	n. d.		
Pro 291	31.95	n. d.		
Pro 300	32.03	26.88	5.16	δ
Pro 313	32.33	n. d.		δ
Pro 323	31.03	n. d.		δ
Pro 331	32.43	27.90	4.52	δ
Pro 347	n. d.	n. d.		
Pro370	31.76	n. d.		
Pro 374	n. d.	n. d.		δ
Pro 381	32.27	n. d.		δ
Pro 384	31.51	27.37	4.14	δ
Pro 386	32.43	27.90	4.52	δ

[a] taken from (Schwarzinger *et al.* 2000)

n. d.: not determined

SI Table 3: NMR statistics

NMR distance and dihedral constraints	
Distance constraints	1170
Total unambiguous NOE	957
Intraresidue	536
sequential (|i-j| =1)	208
short (2<|i-j| <3)	96
medium (4<|i-j| <5)	40
long (|i-j| >5)	77
Total ambiguous NOE	213
Hydrogen bonds	30
Total dihedral angle restraints	185
phi	93
psi	92
Residual dipolar couplings (N-H)	44
Structure statistics	
Violations (mean and SD)	
Distance constraints, Å	0.07±0.01
Dihedral angle constraints, °	1.5±0.2
Max. dihedral angle violation, °	6.1
Max. distance constraint violation, Å	1.0
Ramachandran analysis	
Most favored, %	85.3
Additionally allowed, %	12.7
Generously allowed, %	1.0
Disallowed, %	1.0
Deviations from idealized geometry	
Bonds lengths, Å	0.004
Bond angles, °	0.53
Impropers, °	0.63
Average pairwise rmsd, Å	
Heavy, all	2.8±0.4
Backbone, all	2.6±0.4
Heavy, 2nd structure	0.56±0.05
Backbone, 2nd structure	0.27±0.03

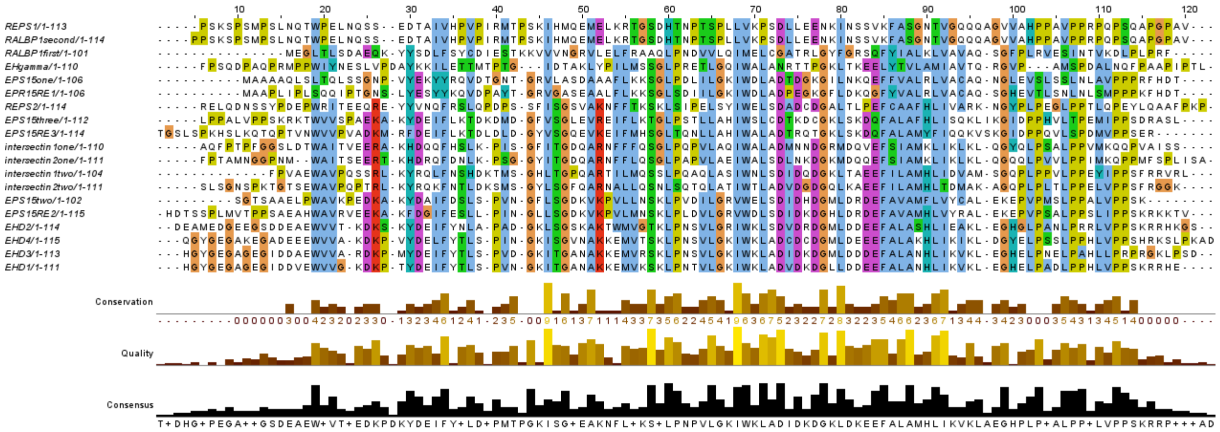

SI Figure 1

Multiple sequence alignment of human EH domain sequences in MUSCLE. Alignment is color coded according to the ClustalX color scheme. The following sequences have been used for the alignment: EH domains from REPS 1 and 2, the first and second EH domains from RALBP1, the first and second EH domains from intersectin 1, the first and second EH domains from intersectin 2, the three EH domains from EPS15, the three EH domains from EPS15RE, the EH domains from EHD1-4 and the EH domain from γ-synergin.

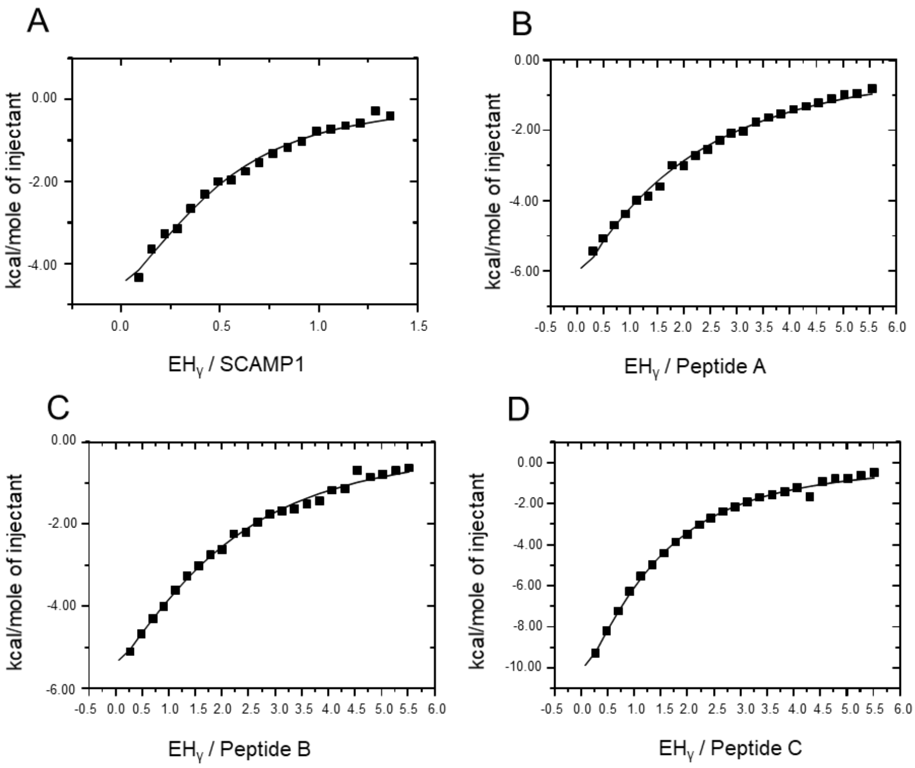

E

	EHγ			
Interaction partner	N	K_D (μM)	ΔH (kcal/mol)	ΔS cal/mol/deg
SCAMP1	0.38 ± 0.04	9 ± 2	-7.5 ± 1.1	-2.3
SCAMP1’	n.b.	n.b.	n.b.	n.b.
SCAMP1’’	0.33 ± 0.01	9.9 ± 0.4	-41.3 ± 1.3	-118.0
Peptide A	1.2 ± 0.1	250 ± 20	-21.1 ± 2.4	-55.2
Peptide B	1.1 ± 0.2	140 ± 20	-12.0 ± 1.5	-23.3
Peptide C	1.0 ± 0.1	90 ± 10	-22.9 ± 2.7	-59.8

SI Figure 2

Thermograms obtained from isothermal titration calorimetry performed on EH*γ* domain and SCAMP1 (A), and peptides A-C (B-D). Integrated heat changes upon binding were plotted against the EH*γ*/interaction partner ratio resulting in differential binding isotherms that were analyzed by a one-site binding model. Resulting binding parameters of all ITC titrations are summarized in (E); n.b. refers to no binding.

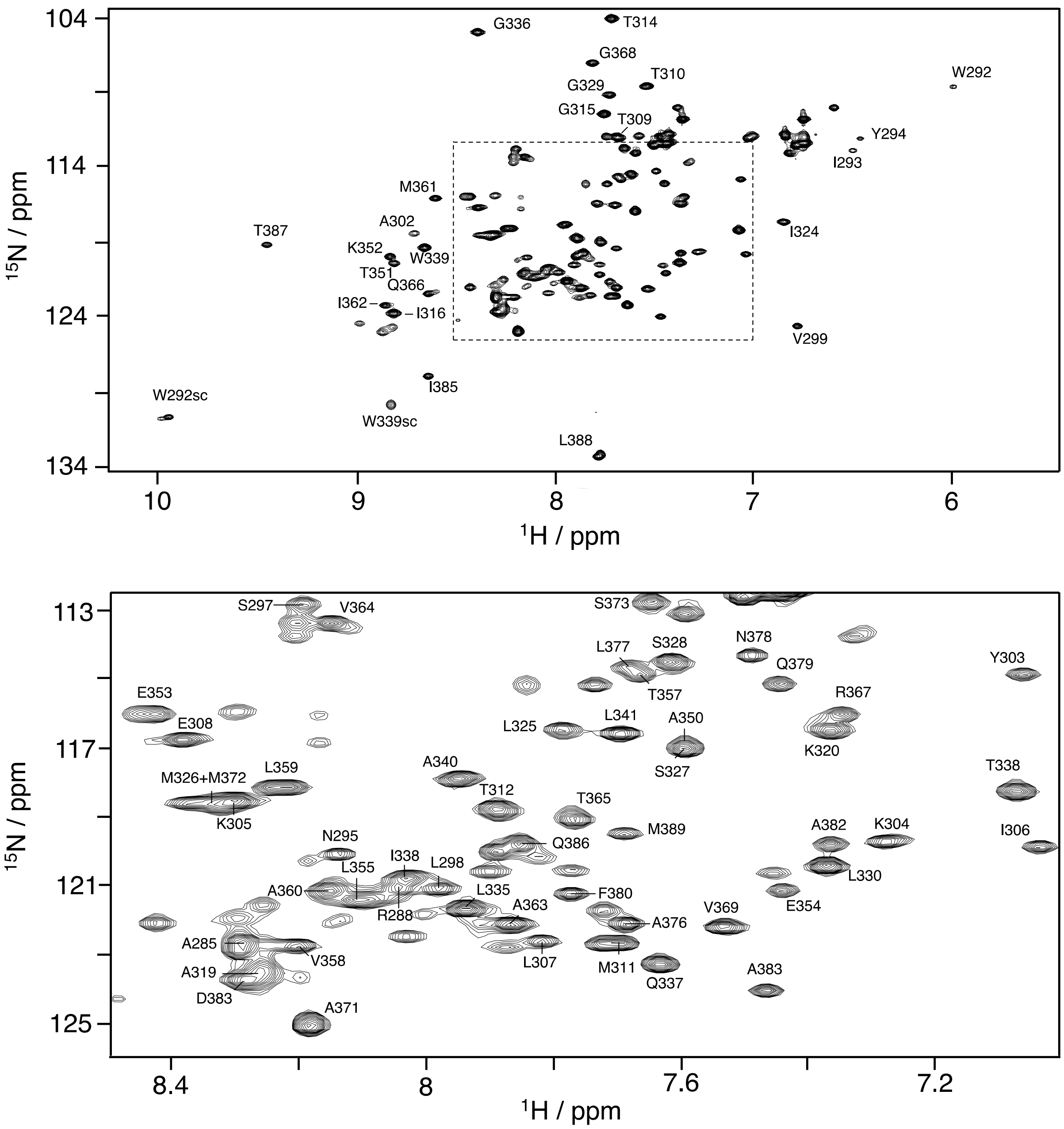

SI Figure 3

Assignment of cross peaks representing EHγ in a heteronuclear two-dimensional ^{1}H-^{15}N HSQC NMR spectrum. Note that assignment of cross peaks located inside the box (dashed line mode) is shown in the lower panel. The one letter code for amino acids has been applied followed by the position in the primary sequence. "sc" refers to side chain. Data have been acquired at T = 298 K and B_0 = 20 T.

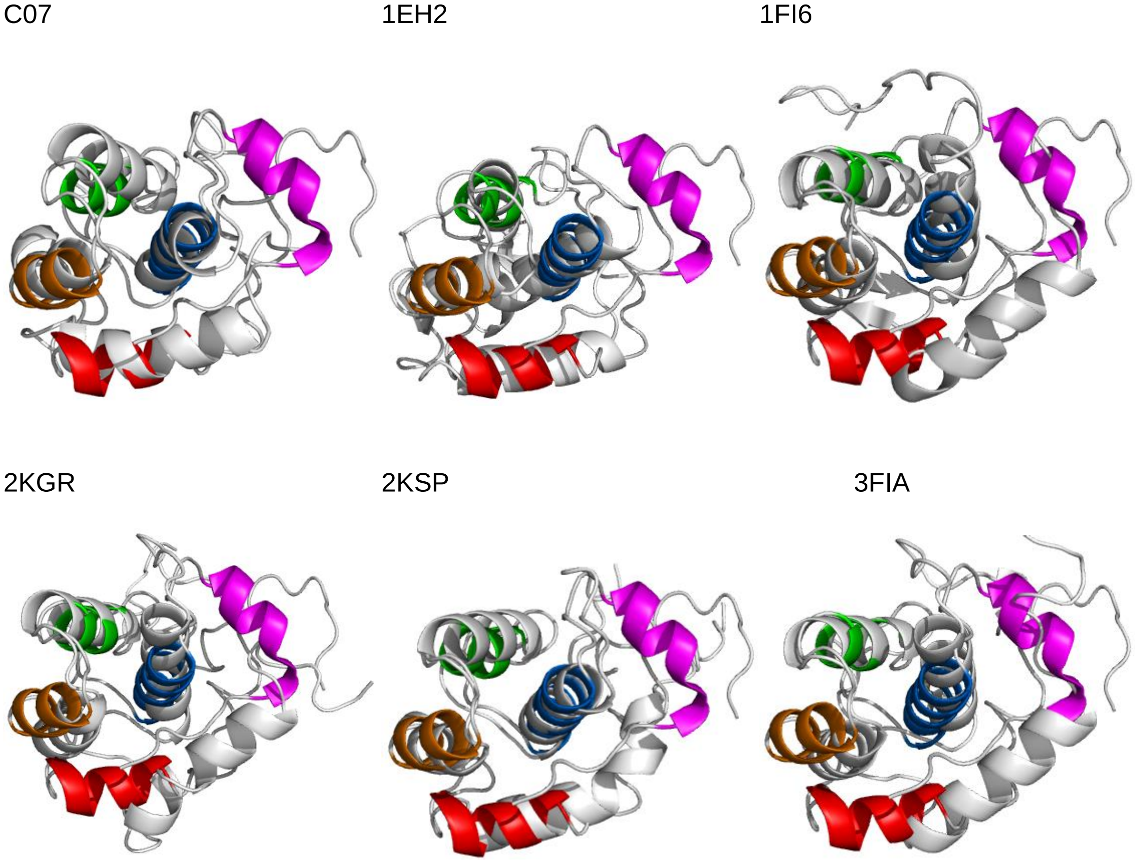

SI Figure 4

Comparisons between the lowest energy structure of EHγ determined in this study (helical elements highlighted in color) to homologues structures (colored in light grey). The following structures are shown: Third EPS15 homology domain of human EPS15 (pdb code 1C07), second EPS15 homology domain of human EPS15 (pdb code 1EH2), EH domain of REPS1 (pdb code 1FI6), ITSN1 from human (pdb code 2KGR), EHD-1 EH domain (pdb code 2KSP) and EH 1 domain from human intersectin 1 (pdb code 3FIA). The alignment has been conducted for backbone atoms comprising helices a and d (1C07), b and d (1EH2, 2KSP), c and d (1FI6, 2KGR, 3FIA).

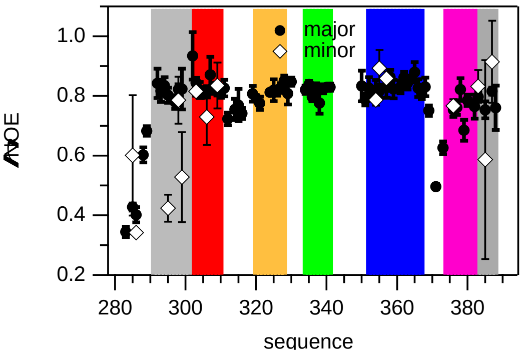

SI Figure 5

Comparison of fast time scale dynamics between signals representing either major or minor conformation of EHγ. Experimental data have been acquired at T = 298 K and B_0 = 20 T by {^{1}H-}^{15}N heteronuclear NOE (*h*NOE) and analyzed for major (filled circles) and minor conformation (open rectangles). Colors used for highlighting the background refer to the structural composition of EHγ domain presented in Fig. 2A. Error bars refer to the standard deviation obtained from three independent measurements.

A

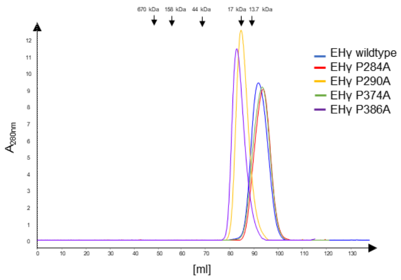

C

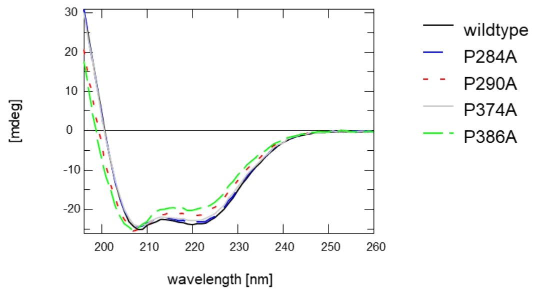

B

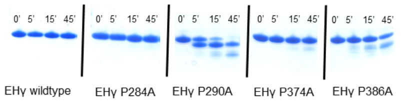

	wildtype	P284A	P290A	P374A	P386A
helical content	43.1%	42.6%	38.2%	42.6%	35.8%
222nm/208nm	0.94	0.95	0.84	0.94	0.79

SI Figure 6

Biophysical characterization of EHγ wild type and mutants. (A) Gel filtration profiles of purified EHγ variants on a Superdex 75 column. EHγ P290A and EHγ P386A elute at a lower retention volume compared to wild type and the other mutants. Molecular weight standards are highlighted. (B) Limited proteolysis of EHγ wild type and mutants in the presence of chymotrypsin highlight that EHγ P290A and EHγ P386A are prone to degradation already at low concentrations of the protease. The time points when the reactions were stopped (in minutes) are shown. (C) Far-UV-CD spectrum of EHγ wild type and mutants recorded at the same concentration. Deconvolution of the data resulting in the helical content and the 222nm/208nm ratio is highlighted below.

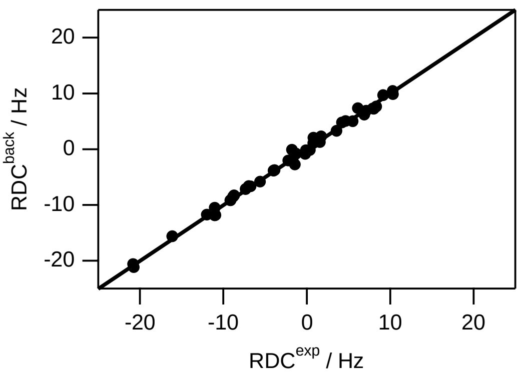

SI Figure 7

Correlation plot between experimentally determined and back-calculated RDCs values determined for EHγ. Back-calculation has been done using 2MX7.pdb applying the structure of EHγ that possesses lowest energy.
